# Digital health literacy, vaccine information sources, and vaccine acceptance among parents in Ontario: Quantitative findings from a mixed methods study

**DOI:** 10.1371/journal.pgph.0003154

**Published:** 2024-05-17

**Authors:** Sarah Ashfield, Lorie Donelle, Panagiota Tryphonopoulos, Ève Dubé, Maxwell Smith

**Affiliations:** 1 Arthur Labatt Family School of Nursing, Faculty of Health Sciences, University of Western Ontario, London, Canada; 2 College of Nursing, University of South Carolina, Columbia, South Carolina, United States of America; 3 Department of Anthropology, Faculty of Social Sciences, Université Laval, Quebec, Canada; 4 Institute National de Santé Publique du Québec, Quebec, Canada; 5 School of Health Studies, Faculty of Health Sciences, University of Western Ontario, London, Canada; University of Michigan, UNITED STATES

## Abstract

Parents make important vaccination decisions for their children and many variables affect parents’ decisions to accept or decline vaccines. Parents are tasked with locating, understanding, and applying information to inform health decisions often using online resources; however, the digital health literacy levels of parents are unknown. The purpose of this study was to investigate parents’ digital health literacy levels, their sources for vaccine information, and analyze how demographics, digital health literacy, health literacy, parental attitudes and vaccine beliefs, trust, and vaccine information sources predict vaccine acceptance. Quantitative findings of a mixed methods study that examined parental vaccine decision making across the continuum of vaccine hesitant to vaccine accepting is reported. An online survey of parents of young children living in Ontario, Canada was conducted in 2022. Multiple linear regression determined predictors of vaccine acceptance. 219 participants completed the survey and on average reported adequate digital health literacy skill. Healthcare providers were reported as the most commonly used source of vaccine information. Two models were retained that predicted vaccine acceptance, both models predicted about 50% of the variability in vaccine acceptance. Model A identified that trust predicted parent vaccine acceptance and model B identified that digital health literacy, and the vaccine information sources healthcare providers, family and friends, and alternate healthcare providers predicted vaccine acceptance. Family and friends and alternate healthcare providers negatively predicted vaccine acceptance. Most parents in our study had high levels of digital health literacy. Opportunities exist for further research and policy change focused on trust at a systemic public health level. While clinical level implications included the importance of healthcare providers as a vaccine information source and adequate digital health literacy to facilitate parental vaccine decision making. Continued efforts to develop awareness on the importance of digital health literacy among the public and healthcare providers is needed, including further research on the digital health literacy levels of Canadians.

## Introduction

Parents are accountable for making important medical decisions for their children, including routine childhood vaccinations and other vaccinations such as influenza and COVID-19. Public health organizations continue to promote the importance of childhood vaccinations, and the COVID-19 pandemic brought further public attention to the critical role that vaccines play in reducing the morbidity and mortality associated with vaccine preventable diseases [1]. Although the majority of Canadians believe that childhood vaccines are safe and effective, vaccine hesitancy continues to be a significant issue surrounding vaccine decision making [2–4]. Even before the COVID-19 pandemic, vaccine hesitancy—the reluctance or refusal to vaccinate despite access to vaccine services—was listed as one of the top 10 threats to global health in 2019 [5]. Vaccine hesitancy has contributed to the resurgence of vaccine-preventable diseases such as measles, polio, and pertussis, despite childhood vaccination being one of the most cost-effective ways to avoid these diseases [5].

Parental vaccine decision making is complex and involves emotional, cultural, social, spiritual, and political factors [6, 7]. A close relationship exists between trust and risk assessment in relation to vaccine decision making, particularly among parents [8, 9]. This risk assessment process involves parents weighing the risk of their child(ren) contracting a vaccine-preventable disease against the negative impact a vaccination could have on their child’s health [6, 9]. Vaccine acceptance can be influenced by trust in many areas including the product (vaccine), the provider (healthcare professionals), the policymaker (health system, government and public health researchers involved in making and recommending the vaccine) and in science and scientific institutions [10–12]. Further to this, trust in pharmaceutical companies, large organizations, government, public health organizations and healthcare providers can impact vaccine risk assessment and decision making [6, 11, 13].

A pre-requisite of vaccine decision making is access to information to inform decisions [7]. Parents access health information as a function of their ability to find, understand, critique, and apply information to inform the health and wellbeing of their family members and self. Health information seeking strategies include face-to-face health teaching from healthcare providers, mass media (e.g., television and radio), printed information (e.g., newsprint, books, pamphlets) and increasingly individuals are searching for and locating information online [14, 15]. The many benefits of online health information resources need to be weighed against the challenges of misinformation, such as unverified information online, and even more concerning is disinformation (purposely harmful information) that challenges individuals’ ability to locate and access reliable evidence-based information to inform their health decisions [16, 17]. Digital health literacy is defined as the ability to seek, find, understand, and appraise health information from electronic sources and apply the knowledge gained to addressing or solving a health problem [18]. According to national digital health literacy survey data, Canadians’ (16–64 years) skills range from problematic to sufficient but on average Canadians have sufficient skill [19]. Younger Canadians (aged16–34 years) were reported to have significantly greater skill than those 35 years and older [19]. Inquiry into parents’ digital health literacy skills is limited, however of concern is that approximately 60% of Canadians lack the health literacy skills to make informed health decisions and manage their health needs [20]. The most recent assessment of American’s health literacy levels demonstrated that at least 88% of adults living in the United States have health literacy levels that are inadequate to navigate the healthcare system [21].

This paper is part of a larger study that investigated parents’ vaccine decisions on the continuum of vaccine hesitant to vaccine acceptance as a function of information seeking patterns, health literacy, digital health literacy, trust, vaccine attitudes and beliefs, and impact of the COVID-19 pandemic. Reported here are the quantitative findings focused on parents’ digital health literacy levels, their sources for vaccine information, and analysis of how demographics, digital health literacy, health literacy, trust, and vaccine information sources influence vaccine acceptance.

## Methods

### Study population and setting

We conducted a cross-sectional, non-experimental study to examine parental vaccine decision making surrounding routine childhood and COVID-19 vaccines. Eligible participants included: parents or guardians (18 years and older) of children aged 2–11 years, English speaking individuals, those who make vaccine decisions for their child(ren), living in the province of Ontario. The survey was available only online and was therefore limited to those with internet access and a technological device to access the survey. Online distribution of the survey was chosen for two main reasons. First, to respect public health guidelines to prevent the spread of SARS-CoV-2 (the virus that causes COVID-19) and second, to access participants known to be present online.

The research questions that informed this study were: 1) What are the health information seeking patterns of parents and what individual factors influence decision making? 2) What are the relationships between the independent variables (digital health literacy, health literacy, measures of trust, parental vaccine attitudes and beliefs, and vaccine information sources) on the outcome variable, vaccine acceptance?

Throughout this study, federal authorization, and provincial guidelines on the use of COVID-19 vaccinations for children were evolving. Vaccination of Canadian children aged 16 years and older against COVID-19 began December 9, 2020, with expansion to those 12–15 years of age on May 5, 2021 [22]. Immunization of children aged 5–11 years of age was authorized on November 19^th^, 2021, with the final extension of the vaccine to children 6 months to 5 years of age on July 14, 2022 [22–24]. Public health social distancing guidelines and various mandates to prevent transmission of SARS-CoV-2 were evolving based on local, provincial, and federal transmission and risk assessments.

### Ethical considerations

This study was conducted in accordance with the Tri-Council policy statement for ethical conduct for research involving humans [25]. Formal implied consent was obtained from the participants prior to completion of the survey. Potential participants were provided information about the study and indicated if they agreed to the following statement: “By completing the following questionnaire you indicate your voluntary agreement and consent to participate in this research study”.

### Recruitment

Participant recruitment was conducted primarily through the social media platform Facebook. Paid Facebook advertisements and recruitment posters were placed within specific Facebook parenting groups. Facebook was chosen as the primary recruitment method for several reasons: 1) previous success of this recruitment method among the specific participant population, 2) to comply with public health restrictions, and 3) evidence that parents are looking online for vaccine information [14, 26]. Recruitment posters that included the survey link were also emailed to various health care agencies, providers, and organizations that parents of young children frequent in west-central, southern, eastern, and northern Ontario. Full details on the recruitment methods, including challenges and successes of this method, are published separately [27]. A calculated minimum required sample size of 204 participants was determined. Cohen’s [28] statistical power analysis using a moderate effect size and power of 0.95 was utilized to calculate the sample size for this study [29].

### Data collection

Data collection began on July 11^th^, 2022, and completed September 30^th^, 2022. The survey was conducted using the Qualtrics survey platform version 2020 [30]. The questionnaire was available online and the average time for completion was approximately 8 minutes.

### Instrumentation

Survey questions focused on participants’ socio-demographics (age, gender identity, relationship to children, ethnicity, education, number of children) and children’s vaccination status, digital health literacy skills, health literacy skills, attitudes and behaviors surrounding childhood vaccines, trust, sources of vaccine information, and vaccine hesitancy or acceptance. Validated instruments were used to measure the independent variables (digital health literacy, health literacy, trust, parental vaccine attitudes and beliefs, and measure of vaccine acceptance) and the dependent variable (vaccine acceptance) and are described in detail below. Parents’ self-reported sources of vaccine information were documented through researcher-generated close-ended questions.

#### Digital health literacy

Digital health literacy was measured using the eHealth Literacy Scale (eHEALS), an 8-item instrument [31]. This scale measures the self-reported ability to find, assess, and use online information to make informed health-related decisions. Each item is scored on a 5-point Likert scale from 1–5 where 1 indicates disagree and 5 indicates strongly agree; scoring ranges from 8–40 with higher scores indicating higher digital health literacy skill [31]. Several studies have utilized a cut-off of 26 and below to indicate problematically low levels of digital health literacy [32, 33]. Internal consistency was previously demonstrated with a Cronbach’s alpha of .94, and construct validity of the digital version of the instrument was determined with exploratory factor analysis with single factor retention based on an eigenvalue of 5.74 [34]. The psychometric properties of this instrument confirm that it is a reliable and valid instrument [34–37].

#### Health literacy

Health Literacy was measured using a modified version of Ishikawa’s Health Literacy Scale [38]. This 13-item instrument measures functional, communicative, and critical health literacy on a Likert scale, with a higher score indicating higher health literacy skill [38]. This instrument was initially developed to measure health literacy in diabetic patients, and has been previously adapted and utilized to assess the health literacy of parents in vaccination studies [38, 39]. The 5 items that measure functional health literacy are reverse coded so that a higher score indicates higher functional health literacy [38]. Psychometric properties of this scale have been previously evaluated demonstrating that it is a valid tool [40]. Reliability has been established in each subscale in previous vaccine hesitancy studies with Cronbach α of 0.70 and 0.90 (functional subscale), 0.66 and 0.81 (communicative subscale) and 0.81 and 0.89 (critical subscale) [39, 41].

#### Parental vaccine attitudes and beliefs

Parental vaccine attitudes, beliefs, and vaccine behaviours were measured via the Parental Attitudes about Childhood Vaccines Survey (PACV) [42]. This scale was designed to identify vaccine hesitant parents who under-immunize their children [43]. The readability of this instrument is rated at a grade 6 level, it can be completed in less than 5 minutes, and contains 15 items with three content domains: vaccine beliefs about safety and efficacy, attitudes about vaccination mandates and exemptions, and vaccination behavior [44]. Scoring is on a scale from 0–100, with higher levels indicating vaccine hesitancy. Stability was previously demonstrated with highly concordant baseline and 8 week follow up scoring on a sample of over 400 parents [43]. Previous evaluations of the psychometric properties of this instrument have confirmed that it is valid and reliable [43, 45, 46].

#### Trust

The Emory Vaccine Confidence Index (EVCI) was utilized to measure trust [47]. This instrument was designed to measure trust among parents making vaccine decisions for their children [47]. The EVCI evaluates three domains of trust including the immunizations themselves, the healthcare providers who administer the vaccines, and the process of regulating and recommending vaccines [47]. These three domains of trust are supported by literature on vaccine acceptance as discussed in this systematic review by Larson and colleagues [11]. This instrument asks about trust surrounding scientists, government agencies that approve and regulate vaccines, vaccine authorizers, and healthcare providers who provide information about vaccines. It contains 8-items with a possible score from 0–24, where a higher score indicates greater trust [47]. Scores from 0–12 indicate low levels of trust, and scores of greater than or equal to13 are considered adequate levels of trust [47]. Reliability has previously been established with a Cronbach’s α of 0.857 [47].

#### Vaccine acceptance

Vaccine acceptance was measured by examining parents’ antecedents to vaccination with Betsch’s 5 C’s instrument [48]. This instrument goes beyond measuring vaccine confidence by also measuring vaccine complacency, constraints, calculation, and collective responsibility as aspects of decision-making regarding vaccination choices [48]. The short 5-item version was used in this study; scores were summed and translated to a score out of 100, with a higher score indicating higher vaccine acceptance [48]. A cut-off of 60 and below was utilized to quantify participants as vaccine hesitant while scores of 61 and above were considered vaccine accepting. Reliability of this scale has been previously established through measurement of Cronbach’s α .71 [48]. This instrument’s development was informed by theoretical models of vaccine hesitancy and has been utilized in measuring vaccine acceptance and hesitancy in a variety of settings [49–51].

#### Vaccine information sources

Questions that asked about participants’ vaccine information sources were assessed using researcher-generated questions. Extent literature examining common sources of vaccine information in vaccine-hesitant and vaccine-accepting parents informed the survey questions [52–55]. The question stated: “Indicate the importance of the following sources of vaccine information in general,” with the response options: “not applicable/don’t use”, “not important”, “neutral, “somewhat important”, and “very important”. Vaccine information sources included: “the internet (searching on Google)”, “social media (Facebook, Instagram, Twitter, What’s App, TikTok)”, “my partner”, “friends and family”, “healthcare providers (doctors, nurses, pediatricians)”, and “alternative healthcare providers (chiropractor, naturopath, osteopath)”. These questions were piloted with parents and modified according to feedback for clarity and meaning.

#### Statistical analysis

Statistical analysis was conducted with IBM SPSS Statistics software version 29.0.0.0. Demographic data was analyzed to provide summary descriptions of socio-demographic data consisting of frequency, mean, and standard deviation measures. Descriptive statistics and participant scores for digital health literacy, health literacy, parental attitudes and beliefs, trust, vaccine acceptance, and vaccine information sources were summarized. Univariate analysis was conducted using 1-way ANOVA with post-hoc Games-Howell analysis to identify the difference between participants’ socio-demographics in relation to digital health literacy levels. Multiple regression analysis was conducted to determine the best linear combination of independent variables (demographics, digital health literacy, health literacy, parental vaccine attitudes and beliefs (PACV), trust, and vaccine information sources) for predicting vaccine acceptance. Independent variables that met the conditions required of multiple regression were included in statistical analysis, assumptions for multiple linear regression were checked and met.

Variable inclusion in the regression model was informed by literature, and correlation analysis of variables. Pearson’s correlation coefficient (r) was used for continuous variables and Spearman rho (r_s_) was computed for ordinal level variables. Univariate analysis demonstrated a high correlation between digital health literacy and health literacy (r = .480, p = .001), which was expected as health literacy is a critical component of digital health literacy [56]. As digital health literacy was of primary interest in this study, it was included the model in lieu of health literacy. The assumptions of observations being independent and independent variables being linearly related to the log were confirmed. Sequential stepwise forward regression modelling was utilized, where the independent variables that were highly correlated with the outcome variable were included first r/r_s_ ≥ .50 (trust, PACV, healthcare provider as a vaccine information source). However, multicollinearity proved to be an issue between the PACV and trust, as demonstrated by low tolerance levels of .348 and .333, respectively [Tolerace cut-off of, 1-R^2^ = .42]. As trust is critically important in vaccine decision making, PACV was removed from the model, and trust was retained [11, 13]. Multicollinearity between trust and healthcare providers as a vaccine information source was problematic when both variables were included in the model simultaneously. Therefore, only one was considered at a time.

The next step in variable inclusion in the model involved including independent variables with moderate correlations. The independent variables education (r_s_ = .318, p = .001), digital health literacy (r = .307, p = .001), and alternate healthcare providers as a vaccine information source (r_s_ = -.400, p = .001) were input into the model. Parents’ education was also included in the model informed by the research literature on its importance in parental vaccine acceptance. This model significantly predicted vaccine acceptance; however, multicollinearity was problematic between trust and healthcare providers as a vaccine information source. Therefore, the model was rerun with each variable separately. Family and friends as sources of vaccine information were also added into the model as parents who are present online often search for other parents’ perspectives. Insignificant variables were removed from the model to create the most parsimonious combination of variables that predicted vaccine acceptance. Analysis was considered statistically significant with the p<. 05 alpha level (two-tailed).

## Results

### Demographics

A total of 219 participants completed the survey; most were female (84.9%, n = 186), 31 years or older (96.3%, n = 211), and none were below the age of 26 years. The majority (88.6%) of participants were White, and 8.3% were from various other ethnic backgrounds. Socio-demographic data can be found in Table 1.

**Table 1:** Participant demographics.

Characteristic	n	%
**Gender Identity**	219	100
Male	26	11.9
Female	186	84.9
Non-binary/3^rd^ gender	2	.9
Prefer not to say	5	2.3
**Age**	219	100
18–25 years	0	0
26–30 years	8	3.7
31–40 years	126	57.5
41 years +	85	38.8
**Relationship to child(ren)**	211	100
Parent	211	96.3
Guardian	5	2.3
Other	3	1.4
**Ethnicity**	219	100
South Asian	7	3.2
East Asian	2	.9
Arab or West Asian	3	1.4
Indigenous	4	1.8
Black	1	.5
Latin or Central American	1	.5
White	194	88.6
Other/Prefer not to say	7	3.2
**Level of Education**	218	100
High School	17	7.8
College	60	27.4
University	76	34.7
Graduate University	65	29.7
**Number of Children in Household (aged 2–11 years)**	219	100
1	93	42.5
2	91	41.6
3	26	41.6
4 or more	8	3.7
**Have your children received their recommended routine vaccinations**?	179	100
All vaccinations	179	81.7
No vaccinations	11	5
Some vaccinations	29	13.2

Participants reported high levels of digital health literacy and health literacy demonstrated by a mean digital health literacy score of 33.03 (SD = 5.64) on the eHEALS and a mean score of 9.5 (SD = 1.9) on the Ishikawa health literacy scale [31, 38]. Most participants (84.3%) were vaccine-accepting, while 15.7% were vaccine-hesitant as measured on Betsch’s 5 C’s scale [48]. Participants demonstrated a moderate level of trust with a mean score of 13.4 (SD = 7.4) on the EVCI scale [47]. Full details of instrument scoring are available in Table 2 below.

**Table 2:** Independent variable findings.

Measure	Mean	Standard deviation	Cronbach’s alpha	Scoring Range
Digital health literacy -eHEALS	33.03	5.64	.93	8–40
Health literacy- Ishikawa’s HL scale	9.50	1.90	.77	2–12
Parent vaccine attitudes and beliefs PACV	28.17	32.62	.95	0–100
Trust EVCI	13.40	7.40	.95	0–24
Vaccine acceptance-Betsch 5 C’s	81.00	18.14	.65	0–100

### Vaccine information sources

**Fig 1:**
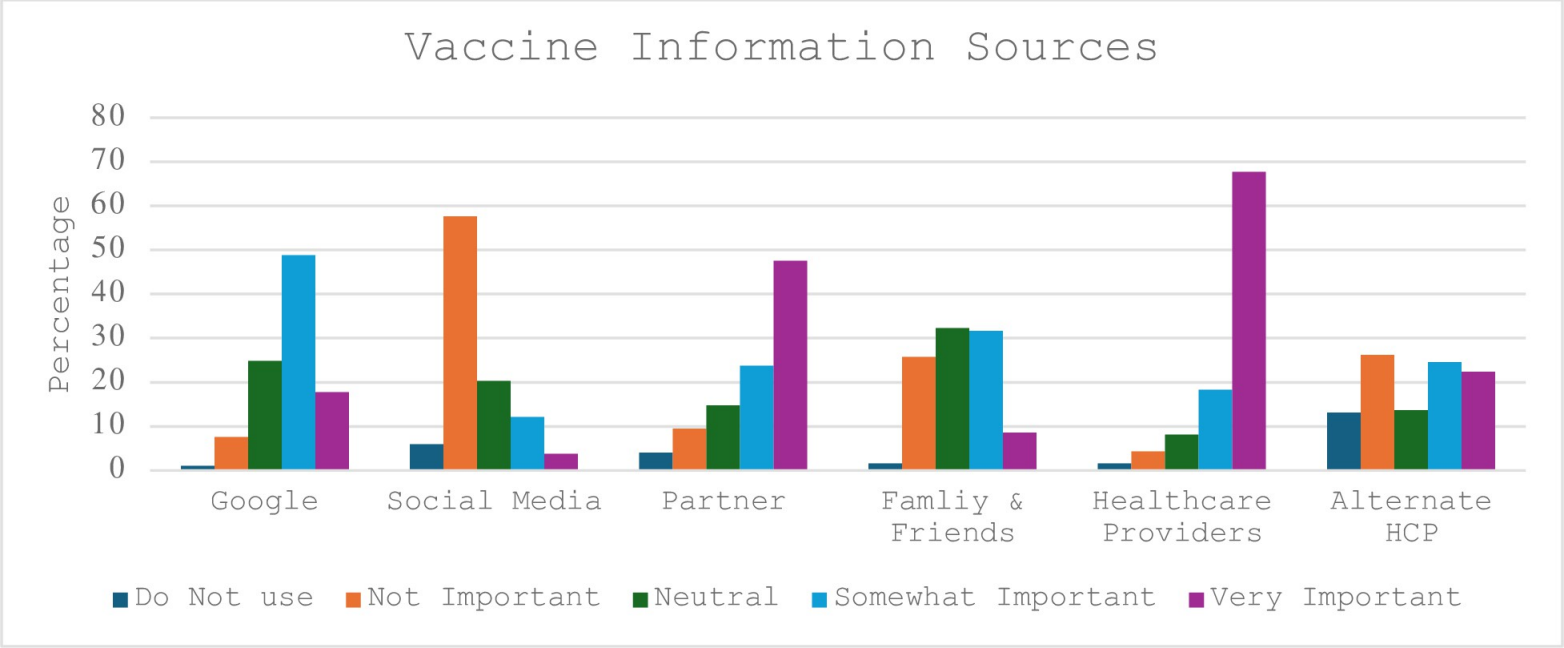
Vaccine information sources.

When participants were asked about sources of vaccine information, 86% of participants rated healthcare providers as somewhat or very important. Alternative healthcare providers–defined as chiropractors, naturopaths, and osteopaths- were identified as somewhat important or very important sources of vaccine information by 47% of participants, and 66% of participants reported searching on Google as somewhat important or very important. Social media was identified as somewhat or very important by 15.9% of participants. A graph of participant vaccine information sources is in Fig 1.

### Digital health literacy

The digital health literacy levels of participants were high ($\bar{x}$ = 33.03, SD = 5.64) measured with the eHEALS instrument. Univariate analysis was conducted to determine if there were statistically significant differences in the digital health literacy (DHL) levels of participants of varying ages, gender identities, number of children, education levels, and children’s vaccine statuses. Parents with graduate-level education had statistically significantly higher levels of self-reported digital health literacy skill compared to parents who had college level education. Parents of children who had received no childhood vaccines had statistically significantly higher levels of digital health literacy skill ($\bar{x}$ = 36.4, SD = 3.9) than parents whose children received some routine childhood vaccines ($\bar{x}$ = 31.54, SD = 6.3). Table 3 displays the statistically significant mean differences found with post hoc Games-Howell test.

**Table 3:** Post hoc Games- Howell–dependent variable DHL.

Variable I	Variable J	Mean Difference (I-J)	p value	95% Confidence Interval	Standard Error
College	University Graduate	-2.88	p = .03**	-5.50, -.22	1.02
Vaccine receipt (some)	Vaccine receipt (none)	-4.83	p = .02**	-8.80, -.60	1.70

** significance at the p = .005 level

### Vaccine acceptance

Using Betsch’s 5 C’s scale, 84.3% (n = 161) of participants were vaccine accepting and 15.7% (n = 30) were vaccine hesitant [48]. In regression analysis, digital health literacy, parents’ education level, trust, vaccine information source-alternate healthcare provider, and vaccine information source-friend and family significantly predicted vaccine acceptance *F*(5, 171) = 35.93, p = .001, with these independent variables accounting for 50% of the variance in the outcome variable of vaccine acceptance (adjusted R^2^ of .498) (model A). However, in model A, only one independent variable, trust statistically contributed to the variance of the outcome variable vaccine acceptance. The coefficients from model A are displayed in Table 4.

**Table 4:** Model A.

Variable	Standardized coefficients	Unstandardized Coefficients		t	Sig
	β	β	Standard error beta (SE β)		
Constant				6.971	p = .001
Trust (in relation to vaccination)	.623	1.518	.161	9.399	p = .001
Digital Health Literacy	.064	.203	.179	1.137	p = .257
Education	.075	1.462	1.146	1.275	p = .204
VIS- Alternate Healthcare Providers	-.045	-.582	.811	-.717	p = .474
VIS-Family and Friends	-.066	-1.200	1.003	-1.196	p = .233

Model R^2^ = .512, Adjusted R^2^ = .498, p = .001

Given the importance of healthcare providers as a vaccine information source in the existing literature on vaccine acceptance, it was important to include in the analysis. Yet, trust and healthcare providers as a vaccine information source couldn’t be included simultaneously in the model due to their multicollinearity. Therefore, an additional analysis was conducted (model B) retaining healthcare provider as an important source of vaccine information; digital health literacy (p = .007), vaccine information source alternate healthcare providers (p = .001), vaccine information source- healthcare providers (p = .001), and vaccine information sources-family and friends (p = .003) significantly contributed to the prediction in vaccine acceptance. Both family and friends and alternative healthcare providers as sources of vaccine information negatively contributed to vaccine acceptance. This combination of variables (model B), reported in Table 5, significantly predicted vaccine acceptance *F* (5, 167) = 30.145, p = .001. The variables in model B account for 46% of the variance in vaccine acceptance, (adjusted R^2^ .459) and while parental education didn’t significantly contribute to (p = .059) vaccine acceptance, it was retained in the final model as it may be clinically relevant.

**Table 5:** Model B.

Variable	Standardized coefficients	Unstandardized Coefficients		t	Sig
	β	β	Standard error beta (SE β)		
Constant				4.322	p < .001
Digital Health Literacy	.159	.522	.191	2.720	p = .007
Education	.114	2.250	1.200	1.900	p = .059
VIS-Alternate healthcare provider	-.196	-2.500	.774	-3.234	p = .001
VIS-healthcare provider	.491	9.140	1.110	8.207	p < .001
VIS-Family & Friends	-.175	-3.180	1.060	-2.993	p = .003

Model R^2^ = .474, Adjusted R^2^ = .459, P = .001

## Discussion

The purpose of this study was to measure parents’ digital health literacy levels, assess their sources of vaccine information, and analyze how the independent variables of demographic information, digital health literacy, health literacy, trust, parental vaccine attitudes and beliefs, and vaccine information sources influenced parental vaccine acceptance. Participants had high levels of digital health literacy; most participants (86%) utilized healthcare providers as a source of vaccine information, and many searched online using the Google search engine (66%) for vaccine information. The use of healthcare providers and Google’s search engine to locate vaccine information is consistent with previous literature on vaccine information sources [53, 54, 57]. Almost half (47%) of parents in our study utilized alternate healthcare providers (naturopaths, chiropractors, and osteopaths) as a source of vaccine information, 40.3% identified family and friends as an information source, and 15% of participants utilized social media as a source of vaccine information.

Our study highlighted two models where several factors predicted vaccine acceptance among parents both models accounted for about 50% of variance in vaccine acceptance. In one instance, trust (in immunizations, healthcare providers, and in vaccine regulation) accounted for about 50% of variance in vaccine acceptance among parents. This model highlights the importance of trust in vaccination at a public health system level, where trust in immunizations, healthcare providers, and vaccine regulation processes’ impact vaccine acceptance.

In the second model participants’ digital health literacy, healthcare providers, alternate healthcare providers, and family and friends as vaccine information sources predicted vaccine acceptance among parents. Healthcare providers as a source of information and digital health literacy positively predicted vaccine acceptance whereas alternate healthcare providers and family and friends as vaccine information resources predicted vaccine hesitancy. Within this model the importance of vaccine information sources and digital health literacy impacted parental vaccine acceptance. This second model has clinical significance where individual level interventions and research can be targeted to understand parents’ vaccine acceptance. It has been established that trust in both relationships with healthcare providers and the vaccine information they provide is crucial for vaccine acceptance [58–60].

### Trust and vaccine acceptance among parents

Our analysis demonstrated that trust is an important factor in vaccine acceptance among parents making vaccine decisions for their children. We specifically measured trust in scientists, government agencies that regulate and authorize childhood vaccines, the vaccines themselves, and the healthcare providers who administer vaccines and supply information to parents. Our findings are consistent with other literature identifying trust in healthcare providers, medical and scientific authorities, and vaccine information sources are important in vaccine acceptance [61–64].

Goldenberg [10] has identified that trust in science and scientific institutions is a crucial component of vaccine hesitancy. In contrast to measuring and evaluating the importance of trust in organizations, government, scientists and institutions, insight into the process of losing trust in various governmental organizations, health authorities, and healthcare providers may provide insight into parental vaccine decisions. Researchers in Finland [65] interviewed vaccine hesitant parents and explored their loss of trust in vaccinations and vaccine related actors or institutions. Parents reported two narratives in losing trust in vaccinations characterized by mistrust or distrust [65]. Participants described mistrust as a gradual cyclical accumulation of knowledge, experiences, and understandings eventually undermining trust in vaccination; where distrust was a linear experience where specific events or experiences started a chain of events leading to loss of trust in vaccination [65]. This research into loss of trust in vaccination further highlights that trust at the systemic level is complex, and that public engagement with science and experts is one part of the process of building trust [65]. Understanding the process of lost trust can provide detail and contextualize insight into what contributes to parental vaccine hesitancy [65].

In Ontario, most vaccines services are provided through primary care [66]. Systemic changes should be focused on regular access to a primary care provider to foster trust between parents and healthcare providers, who provide vaccine counselling and administer vaccines. The province of Ontario is currently experiencing a primary care crisis where over 2.2 million Ontarians are without a primary care provider [67]. This crisis is not unique to Ontarians, as one in six Canadians do not have access to a primary care provider [68]. Having access to a regular primary care provider is critical in establishing and maintaining a trusting relationship. Investing in creative system level solutions to increase access to primary care providers would assist in establishing these relationships. One potential solution would be to expand provincial funding models to allow nurse practitioners to work to their full scope of practice as fully independent primary care clinicians. Research demonstrates that nurse practitioners provide high quality primary care and excel at communication [69]. Literature surrounding trust and nurse practitioners focuses on interdisciplinary collaboration, further research on trust between the patient and nurse practitioner relationship is needed.

Consideration for enhancing education surrounding immunizations in general and the process of vaccine regulation and recommendations may help enhance trust. One public health focused solution could be incorporating education about vaccines and their importance in mitigating infectious disease into the established provincial elementary and high school educations systems. Nurses working within public health could provide education in the classroom environment regarding the process of vaccine development and regulation information. Incorporation of early vaccine education into the education curriculum is supported by literature on vaccine literacy [70]. Beyond educating children about immunizations and the process of vaccine regulation, Ratzan highlights the importance of supporting vaccination as the social norm from an early age, where getting vaccinated is part of routine health practices [70]. Several Ontario nursing organizations are collaborating on developing and implementing a school health nurse initiative planned to be a long-term policy change [71]. This initiative was born out of the pandemic and is aimed at targeting health inequities and places public health nurses in key positions to provide vaccination literacy education.

### Vaccine information sources

Vaccine information sources impacted parental vaccine acceptance; healthcare providers predicted vaccine acceptance while family and friends and alternate healthcare providers (chiropractors, naturopaths, and osteopaths) were associated with vaccine hesitancy. The number of participants in our study who utilized alternate healthcare providers as a source of vaccine information is high in comparison to the number of Canadians who use alternate healthcare providers in general. In a recent study evaluating Canadians’ use of complementary and alternate providers researchers reported that 42% had used a chiropractor, 11% had used a naturopath, and 8% had used an osteopath in their lifetime [72]. An association between vaccine hesitancy or vaccine refusal and the use of alternative healthcare providers has been reported in the literature [73, 74]. This was consistent with the findings in the current study where alternate healthcare providers as vaccine information sources aligned with parents’ vaccine hesitancy. Yet, the number of participants in our study who are utilizing alternate healthcare providers as a vaccine information source (47%) is higher than the number of those who are vaccine hesitant (16%).

There is limited literature that looks at the role of alternate healthcare providers and their role in providing parents vaccine advice. Busse and colleagues reported that parents in Ontario who endorse a naturopathic doctor as their primary source of vaccine information are more likely to partially, or fully avoid vaccination of their child(ren), and that some parents seek out alternate healthcare providers due to vaccination conflict with their family physician or pediatrician [75]. However, researchers reported that naturopaths in Quebec encouraged clients to make informed decision about vaccination and only engaged in vaccination discussions upon their clients’ request [76]. Filice et al. [77] conducted a content analysis of professional guidelines, recommendations, and discourses on vaccination among chiropractors, naturopaths, and homeopaths. Authors identified complex and diverging views on vaccination that included discussions on vaccine effectiveness, safety, political justifiability, and compatibility with complementary and alternative philosophical and professional boundaries [77]. Further details into what type of alternate healthcare providers parents seek information from, what prompts them to seek out alternate healthcare providers, the type of information shared, and how this information is being shared is needed to further understand the clinical importance of alternate healthcare providers in parents’ vaccine decision making.

### Digital health literacy

This is the first study in Canada to report on the digital health literacy (DHL) levels of parents making vaccine decisions for their children. Participants in our study had high levels of digital health literacy as did parents in a recent study also conducted in Ontario that investigated the use of mobile technologies among new parents during the COVID-19 pandemic [78]. Parent participants in our study reported higher digital health literacy levels than the average Canadian, as assessed in the 2020 Canadian Digital Health Survey (DHS) [19]. Our online recruitment strategy and data collection methods likely elicited a group of participants with high computer literacy skills as these skills are required to use a technological device and navigate the internet to participate in our survey. Previous research has demonstrated that individuals with high DHL have greater access to the internet and computers than those with lower DHL [79]. Among other variables, the digital health literacy levels of parents predicted vaccine acceptance. Clinical implications include the importance of parents being able to locate, understand and apply vaccine information to the vaccine decisions they make for their children. Making not only the vaccine information sources important but the literacy skills to understand and apply information to health decisions including vaccination decision making. Those providing vaccine information and education should be aware of parents’ digital health literacy skills and provide online resources targeted to their skills.

Most participants in our study (92%) had post-secondary education, that aligns with the necessary traditional literacy skills (reading, numeracy, understanding prose) to obtain this level of education. We were unable to compare the DHL skills of our participants to other studies that recruited participants through Facebook, as these researchers did not measure or report on these findings [26, 80–82]. Further measurement of the DHL levels of Canadian parents is needed to determine whether this study’s participants accurately reflect the broader parent population in Canada.

There are many opportunities for future research including research into the role that alternate healthcare providers have in vaccination counselling and how that impacts parents’ vaccine decisions. While literature identifies an association between vaccine hesitancy and the use of alternate healthcare providers, there is an identified gap in the literature on what vaccine information is provided and how parents utilize this information from alternate healthcare providers to inform vaccine decisions for their children. Further research is needed to explore communication and messaging about vaccines between alternate healthcare providers and parents. Opportunities exist for research that looks at what prompts parents to seek vaccine information from alternate healthcare providers, what information is provided, and how parents utilize this information in making vaccine decisions. Research on the moderator effect of trust in vaccine decision making may assist in further understanding of the relationship between trust and vaccine acceptance among parents. Exploration of the trust that parents have in scientists, government agencies that regulate and authorize childhood vaccines, as well as the process of losing trust may provide further details in understanding this construct.

Further research among parents with low digital health literacy levels is critical, as health information continues to be presented in digital formats. Research into parents’ health literacy and digital health literacy using online and offline recruitment and data collection strategies is needed. Having public health and vaccine experts involved in delivering vaccine information to parents in an interactive digital format should be prioritized. AI platforms could be utilized to create an interactive digital platform that could be programmed to provide accurate, up to date, plain-language health topic education and provide real-time answers to vaccine questions.

## Limitations

There are some limitations to our findings. This study was conducted in English only, therefore doesn’t represent individuals living in Ontario who do not speak English. This research was conducted online, with participants recruited primarily through social media. Other data collection methods, such as mail, or phone surveys, may yield different results. The decision to collect data using an online survey reflected time and cost efficiency and adherence to social distancing and public health restriction in place at the time. The homogeneous nature of participants in our study (white, educated, female) may reflect the data collection methods. Other researchers have identified that racialized individuals and those with lower formal education may not be well represented in online surveys [83]. Most participants in this study were recruited through the social media platform Facebook. Findings from this study should not be generalized beyond participants who have been characterized in this study. This study was conducted during the COVID-19 pandemic when guidelines on COVID-19 authorization for children were evolving. The environmental context of the pandemic, vaccine information dissemination online and through traditional media channels, as well as concern over protection of children from the SARS- CoV2 virus may impact the perspective of participants.

## Conclusion

This study identifies that parental trust (in vaccines, among the healthcare providers who administer and provide vaccine counseling, and the processes of vaccine regulation), parents’ digital health literacy, and healthcare providers as a source of vaccine information influence vaccine acceptance. In contrast, alternate healthcare providers and family and friends as vaccine information sources were associated with vaccine hesitancy. This research identifies factors that address parental vaccine acceptance at both the clinical and health systems levels. Efforts to foster trust at the public health systems level as well as clinically on an individual parental level is needed.

This study highlighted the importance of trust at a systems level within various organizations and institutions and the processes involved in vaccine regulation in an effort to encourage vaccine acceptance. Further research into the lack of trust in various government organizations, healthcare authorities, public health institutions, scientists involved in making vaccines, and those that regulate and authorize vaccines is needed to understand how this impacts parents who make vaccine decisions for their children. Continued efforts to develop awareness among the public and healthcare providers on the importance of digital health literacy and its impact on health decision making are critical.
